# Environmental Tobacco Smoke Exposure at Home and High-Sensitivity C-Reactive Protein Levels in Three-to-Five-Year-Old Children

**DOI:** 10.3390/ijerph14101105

**Published:** 2017-09-23

**Authors:** Eunkye Kang, Soo Young Kim, Seong Sil Chang, Sinye Lim, Hwan-Cheol Kim, Chul-Gab Lee, Yu-Mi Kim, Su Young Kim, Kee-Jae Lee, Suejin Kim, Mina Ha

**Affiliations:** 1 Department of Occupational and Environmental Medicine, Eulji University Hospital, Daejeon 35223, Korea; anne1727@hanmail.net (E.K.), kimsooy@gmail.com (S.Y.K.), sc2007@gmail.com (S.S.C); 2 Department of Occupational and Environmental Medicine, College of Medicine, Kyung Hee University, Seoul 02447, Korea; drforest@hanmail.net; 3 Department of Occupational and Environmental Medicine, School of Medicine, Inha University, Incheon 22332, Korea; carpediem@inha.ac.kr; 4 Department of Occupational and Environmental Medicine, College of Medicine, Chosun University, Gwangju 61453, Korea; eecg@daum.net; 5 Department of Preventive Medicine, College of Medicine , Dong-A University, Busan 50612, Korea; kimyumi@dau.ac.kr; 6 Department of Preventive Medicine, Cheju Halla University, Jeju 63585, Korea; suy0202@jejunu.ac.kr; 7 Department of Information and Statistics, Korea National Open University, Seoul 03087, Korea; kjlee@knou.ac.kr; 8 Environmental Health Research Department, National Institute of Environmental Research, Incheon 22689, Korea; suenier@korea.kr; 9 Department of Preventive Medicine, College of Medicine, Dankook University, Cheonan 31116, Korea

**Keywords:** environmental tobacco smoke, hs-CRP, preschool children, cardiovascular risk factors

## Abstract

Exposure to harmful environmental factors is particularly detrimental to younger children. We investigated the relationship between environmental tobacco smoke (ETS) exposure in pre-schoolers at home and the level of high-sensitivity C-reactive protein (hs-CRP), a predictive factor for cardiovascular disease. This study was conducted in 2014 and was based on the data of preschool children from the Korean Environmental Health Survey in Children and Adolescents (2012 to 2014), a nationally representative sample. Of 577 children, aged three to five years, 482 were eventually selected for the analysis after excluding those with missing variables, or whose hs-CRP level exceeded the reference value. The proportion of pre-school children exposed to ETS at home was 14.8%. The odd ratios (OR)s of hs-CRP > 1mg/L were 4.90 (95% Confidence Interval (CI) = 1.04–23.17) and 11.66 (95% CI = 1.90–71.65) in the groups exposed to ETS 3–4 times and ≥5 times daily, respectively, compared to the non-exposed group. The risk of elevated levels of hs-CRP showed an increasing trend proportionally to the increase in ETS exposure frequency (*p* for trend = 0.03). Anti-smoking educational programs for parents and guardians may be helpful to reduce ETS exposure at home.

## 1. Introduction

Although the prevalence of environmental tobacco smoke (ETS) varies depending on the operational definition of exposure and participants’ age, the World Health Organization (WHO) reported that nearly 60% of children aged 3−11 years were still exposed to ETS in 2006 in the United States [1]. Another recent study from the Center for Disease Control and Prevention (CDC) showed that, while the prevalence of ETS exposure is declining, it is still greater than 40% as reported from 2011 to 2012 in US children aged 3−11 years [2]. According to a retrospective analysis of data from 192 countries, 40% of children aged 0–14 years were exposed to ETS, which was higher than the proportion of female and male non-smokers (35% and 33%, respectively). Furthermore, the exposure source was mostly their parents [3]. ETS exposure contributes to the worldwide burden of disease, not only in adults but also in children.

ETS is used as exposure to the substance of the tobacco produced by smoking [4]. ETS includes both secondhand smoke (SHS) which is unwanted inhalation of smoke and thirdhand smoke (THS) which is exposure of residual tobacco smoke pollutants [5]. The main component of ETS is side-stream smoke, the toxic concentration of which is higher than that of mainstream smoke, and the particles of which are small enough to penetrate deeper into the body [4,6]. The adverse health effects of ETS exposure were first reported in 1928, and scientists and doctors became concerned about the potential risk of exposure subsequently [3]. According to a report from the WHO, the harmful effects of ETS on health was first reported in 1950, and the effects of ETS exposure on the health of children and pregnant women have been studied since the 1960s [1].

Children, unlike other age groups, are more vulnerable to ETS exposure at home [7]. Compared to adults, children are more likely to be affected by exposure to ETS at the same concentration since they inhale air at relatively higher ventilation rates [4]. In addition, the younger the children are, and the longer they spend time at home, the more they are exposed to ETS. Moreover, a child experiencing parental smoking is more likely to become a future smoker [8].

The causal relationship between exposure to ETS and health effects has been well established in children as well as in adults. It is well known that exposure to ETS is associated with various types of cancer, pulmonary disease, and cardiovascular disease in adults [1,9]. Exposure to ETS also increases morbidity and mortality related to pulmonary disease in children [3], and aggravates symptoms of diseases such as asthma [10]. In addition, children are vulnerable to immunological disorders, such as atopy or eczema, as a result of ETS [11] as well as neurodevelopmental consequences [12]. Although ETS exposure results in cardiovascular disease mainly in adults, prior studies have shown that long-term exposure to ETS in children is associated with increased risk of early-onset cardiovascular disease [6].

High-sensitivity C-reactive protein (hs-CRP) is detected using advanced techniques of high-sensitivity assays, and its elevated level from the reference range predicts an increased risk of cardiovascular disease. An elevated hs-CRP level is recognized as a risk factor for cardiovascular disease in children as well as adults [13,14,15]. Furthermore, previous studies have shown a relationship between ETS exposure and serum hs-CRP levels in children [16,17]. These studies were, however, mainly focused on school-aged children. There have been few studies focusing on preschool children, infants, and toddlers, in particular, who spend a lot of time at home with parents who are smokers, and may have longer exposure times and higher exposure levels to ETS. A recent cross-sectional study on 139 children aged 2−5 years showed that high cotinine levels were associated with an increase in various cardiovascular risk factors, including hs-CRP, a finding which has been confirmed in adults [18].

In the Korean National Health and Nutrition Examination Survey III (2005–2007), 37.8% of children aged under six years were reported to have been exposed to ETS [8]. The aim of this study was to investigate association between ETS exposure at home and hs-CRP level in Korean preschool children aged between three and five years using a nationally representative, relatively large, and recent dataset.

## 2. Materials and Methods 

### 2.1 Study Population

This study was conducted using the data of the Korean Environmental Health Survey in Children and Adolescents (KorEHS-C), conducted by the National Institute of Environmental Research with the aim of establishing children’s environmental health policy in Korea. KorEHS-C presents the national and regional representative survey data of children’s environmental exposure and health status (2012–2014). The participants of the survey were children aged 6−18 years from 2012 to 2013, and children aged 3−5 years in 2014 [19]. The present study used the survey data of preschool children aged three to five years in 2014. The number of participants was 577 at 63 preschool institutions, such as day care centres or kindergartens, which were randomly selected based on a stratified two-stage cluster sampling design. In the first sampling stage, preschool institutions were sampled with probabilities proportional to their size from the list of all preschool institutions. At each sampled preschool institution, nine participants were randomly selected by stratified sampling according to age and sex [20].

Considering the participants’ age group, the questionnaires on environmental exposure assessment and health items were completed by parents or guardians. Examiners visited the institutions and conducted physical examinations and collected bio-specimens for blood tests. Of the 577 participants, 482 participants (83.5%) were eventually selected for the analysis after excluding children with missing information on ETS exposure in the questionnaire, those without biological samples (*n* = 86) and those whose hs-CRP level was higher than 10 mg/L (*n* = 9), which was the reference level for abnormality [21]. The excluded children showed no significant differences in general characteristics compared to those included in the present study, except for age where slightly older children were excluded (Table S1).

The study protocol, including questionnaires and consent forms for assessment, were approved by the Institutional Review Board of Dankook University Hospital (IRB number: DKUH 2014-06-007-002). Written consent was obtained from participants’ parents or guardians before enrolment.

### 2.2 ETS Exposure at Home

The participants were defined as being exposed to ETS at home if their parents or guardians answered “yes” to the question, “Does your child smell cigarette smoke from others at home?” The ETS exposure variable was categorized to “yes/no”. They were further categorised into three groups according to the frequency of ETS exposure per day from answers to the question “How many times a day does your child smell the cigarette smoke?” (1–2 times, 3–4 times, and ≥5 times).

### 2.3 Serum High-Sensitivity C-Reactive Protein

In order to measure the hs-CRP level, blood samples were collected from children after an overnight fast. Five milliliters of sample was collected using Vacuum Tube SST/Grei, and was analyzed by a turbidimetric immunoassay (TIA) using Cobas 8000-C702 in a commercial laboratory. The limit of detection was 0.1 mg/L and the coefficient of variations ranged between 2.06% and 3.92%.

Based on the guideline for serum hs-CRP level from the American Heart Association (AHA) and CDC, we adopted 1.0 mg/L as a reference value for low risk of cardiovascular disease and categorized the participants into two groups: low (≤1 mg/L) and high (>1 mg/L) groups according to adult standards. The samples of hs-CRP level >10 mg/L were excluded, since it was recommended that the samples be retested two weeks later [21].

### 2.4 Potential Confounders and Covariates

Anthropometric measurements were performed after checking whether participants suffered from any infectious disease or febrile illness at that time. The weight (in kilograms, kg) and height (in centimeters, cm) of all participants were measured using identical devices and the body mass index was calculated based on the standard formula and expressed as kg/m^2^. The HEM-907 (OMRON, Tokyo, Japan) was used to measure the blood pressure in preschool children, based on the recommendations of the European Society of Hypertension for blood pressure measurement in adolescents. The blood pressure was measured twice after five minutes of seated rest. For the history of asthma and atopic dermatitis, the participants were categorized into “yes” or “no” groups according to answers to the question, “Has your child been diagnosed with the aforementioned diseases by a doctor?” For the socioeconomic status of participants’ families, they were categorized into five groups according to the household income: “upper class”, “upper of middle class”, “middle of middle class”, “lower of middle class”, and “lower class”.

### 2.5 Statistical Analysis

T-test, generalized linear regression model, χ^2^ test, and Kruskal–Wallis tests were used to test for differences between groups. Multiple logistic regression adjusted for potential covariates was performed to examine an association between prevalence of having a high level of hs-CRP and ETS exposure at home. The regression model on the association between ETS and hs-CRP was adjusted for age, gender, body mass index, white blood cell count (as an indicator of acute inflammation), history of asthma, and history of atopic dermatitis as covariates in the multivariate models. The *p* for trend was calculated using the continuous scale of the categorical variables in the corresponding model. All analyses were considered in the complex sampling design. SPSS (version 18.0 for Windows, Chicago, IL, USA) was used and the significance level was 0.05.

## 3. Results

### 3.1 General Characteristics of Participants

The prevalence of exposure to ETS in preschool children at home was 14.8%, and 1.4% were exposed more than five times a day. No significant difference in ETS exposure frequency by children’s age was observed. The proportion of children having a history of asthma and atopic dermatitis were 6.0% and 29.4%, respectively. Children who had a history of atopic dermatitis showed a significantly higher prevalence of ETS exposure than those who did not. There were no significant differences with respect to sex, history of asthma, and level of household income (Table 1).

There were no significant differences between the groups exposed to ETS and the groups that were not, with respect to body mass index, blood pressure, blood glucose level, total cholesterol, white blood cell count, and hemoglobin, as shown in Table 1.

### 3.2 Distribution of hs-CRP

The distribution of hs-CRP was skewed right, therefore, the median (interquartile ranges, IQR) as well as the arithmetic mean (range) were presented. The arithmetic mean (median) of hs-CRP level in all participants was 0.81 (0.38) mg/L. The hs-CRP level was not significantly different between ages, sexes, exposure/non-exposure to ETS, history of asthma or atopic dermatitis, and household income levels. The hs-CRP level tended to be higher according to increases in the daily frequency of ETS exposure, but this was not statistically significant, as shown in Table 2.

### 3.3 Association between ETS and hs-CRP

An elevated hs-CRP level was associated with exposure to the ETS at home, however, it was not statistically significant even after adjusting for covariates. However, a significant increase in the prevalence of elevated hs-CRP levels was observed with an increase in the daily frequency of ETS exposure, in a dose–response manner (*p*-trend = 0.03). In the fully adjusted model, the prevalence rate ratios of having a high hs-CRP level (95% Confidence Interval (CI)), were 4.90 (1.04–23.17) and 11.66 (1.90–71.65) in children exposed to ETS daily at home 3–4 times and ≥5 times, respectively, compared to those who were not exposed (Table 3).

## 4. Discussion

We found that the prevalence of elevated hs-CRP level in preschool children aged three to five years increased according to increases in daily ETS exposure frequency at home in a dose-response manner.

A previous study has reported that exposure to ETS affects the causative factors of cardiovascular diseases, as metabolic syndrome, in children as well as adults [22]. A meta-analysis on 19 published studies showed that the relative risk of cardiovascular disease in adults exposed to ETS was 1.30 (95% CI = 1.22–1.38) [23]. A study on the risk of atherosclerosis in young adults in a birth cohort of 732 participants who were exposed to ETS revealed that permanent vascular damage was caused at an early age [24]. Exposure to ETS leads to hs-CRP elevation by inflammatory responses in the process of vessel damage in children as well. A study by Earl S. et al., using The National Health and Nutrition Examination Survey (NHANES) data indicated that hs-CRP level is related to risk factors for cardiovascular disease in children aged 3−17 years [13].

To the best of our knowledge, this is the first study to examine the ETS exposure at home in association with hs-CRP levels in children aged five years or less. Previously, a study in children and adolescents aged 6–18 years from the NHANES data reported that the hs-CRP levels became elevated by being exposed to ETS [25], which is consistent with the findings of the present study in children aged three to five years.

In terms of hs-CRP level in youths, the median CRP level in 699 British children was 0.15 mg/L (IQR, 0.06 to 0.47 mg/L) [14], the mean hs-CRP level in 79 Finnish children was 0.7 mg/L (range, 0 to 8.6 mg/L) [26], and the median hs-CRP level in male and female children from the third NHANES data, were 0.3 mg/L and 0.4 mg/L respectively [13]. In a study conducted in Korea, the mean hs-CRP level was 1.40 mg/L in the obese group, whereas it was 0.55 mg/L in the healthy control group [27]. In the present study, the median hs-CRP level in children aged 3–5 years was 0.37 mg/L (IQR, 0.24–0.68), whereas the median hs-CRP level in the group exposed to ETS at home was 0.39 mg/L (IQR, 0.23–0.78). The difference in the hs-CRP level among the other countries might be due to differences in participants’ age, which makes it difficult to compare hs-CRP levels directly.

Since atherosclerosis may start in childhood, hs-CRP is associated with the risk of early onset of cardiovascular disease in children [6,28]. A study on healthy children without obesity or metabolic syndrome presented a significant correlation between carotid artery intima-media thickness and hs-CRP level, suggesting that a high hs-CRP level is associated with the initial process of atherosclerosis [26]. Exposure to ETS may initiate an increment of inflammatory substances involved in the formation of atherosclerotic plaque by aggravating vessel damage in children as well as adults. A continuous increase in the level of hs-CRP can be an early warning sign of cardiovascular disease.

The pathophysiology of the effects of exposure to ETS in children has not been clarified yet. Despite the fact that children have a larger amount of inspiratory air per unit of body weight than adults, immature enzyme and detoxification processes in children slow down the metabolism related to toxicity [7]. There are some hypotheses on the mechanism of increased risk of cardiovascular disease associated with ETS exposure in children. Tobacco smoke consists of more than 4000 complex substances, including toxicants and carcinogens. Among these substances, 7,12-dimethylbenz(a,h)anthracene and benzo(a)pyrene, which belong to the chemical class of polycyclic aromatic hydrocarbons, damage the vascular smooth muscle directly, causing endothelial abnormalities [29]. A previous study in healthy children aged 9 to 13 years showed that oxidative injury produced inflammatory substances [30]. Eventually, vascular injury and lowered endothelial function due to increased oxidative stress affected the vessels in children. It is thought that exposure to ETS interrupts the lipid profile, causing acceleration of atherosclerotic plaque formation [31]. In summary, inflammation occurs when exposure to ETS damages the vessels in children, which is presumed to be associated with an elevation of hs-CRP level [31]. Another hypothesis is that platelets, involved in the normal process of hemostasis, inappropriately aggregate as a result of ETS exposure [29]. Coagulation of platelets causes atherosclerosis not only by vessel wall injury, but also by plaque formation through thrombosis. A significantly reduced number of platelets was found after exposure to ETS, and it is presumed that nicotine, carbon monoxide, and other toxic substances derived from cigarette smoke impinge on platelet susceptibility [29,32]. Notably, susceptibility of platelets is affected even in short-term exposure, and the platelet aggregation in non-smokers exposed to ETS was found to increase in the same proportion to that in smokers [23]. Therefore, exposure to ETS affects endothelial injury in a dose-related manner [33].

According to Articles 14 and 15 of the Environmental Health Act in Korea [34], the Minister of Environment must investigate and assess the effects of environmental exposure on the health of susceptible populations such as children, elderly individuals, pregnant mothers, etc. Children spend a lot of time at home. The younger they are, the longer they tend to stay at home. Since children spend much time closely with their parents or guardians, some of whom are smokers, home is the place where many children are usually exposed to ETS. It is difficult to compare the prevalence of ETS exposure in children of different ages due to differences in the age groups observed in different studies. Furthermore, various factors such as tobacco control measures, educational activities, and the method used to measure the exposure (i.e., by means of a questionnaire, nature of the questions, and biochemical verification of exposure) make it difficult to compare studies accurately.

Nonetheless, according to the NHANES III, in which the age group of the participants was similar to that in this study, 40% of children aged less than five years were exposed to ETS in the United States [35]. The data from Canada showed that 37–39% of infants and preschool children lived with smokers in their home [36]. In the Korean National Health and Nutrition Examination Survey III (2005–2007), 37.8% of children aged less than six years were reported to have been exposed to ETS [8]. In the present study (2014), the proportion of preschool children exposed to ETS (14.8%) was relatively lower than that in previous studies. Exposure to ETS seems to have been reduced in recent years due to global and national efforts, such as legislations that restrict smoking to certain areas. In spite of this, some preschool children are still exposed to ETS at home due to difficulties in applying the law within private homes. Increasing awareness by educating parents and guardians would be an effective strategy to reduce ETS exposure. Furthermore, there was a trend towards an increase in the percentage of children exposed to ETS with a decrease in household income in the present study, although not significant. Higher exposure to ETS in children of families with lower socioeconomic positions has been consistently reported in Korea [37] as well as in a European country [38]. Special attention is needed for children of low-income families in the development of public health policy to reduce ETS exposure.

Although the present study did not show a significant result using the binary variable on exposure to ETS, the prevalence rate ratio was significantly increased in the heavily exposed individuals in a dose–response manner in the present study, suggesting an effect of cumulative ETS exposure on hs-CRP level elevation. Previous studies have shown that an accumulation of exposure to cigarette smoke had an impact on the risk factors for cardiovascular disease, showing that five-year changes in carotid atherosclerosis had a dose–response relation with cigarette smoke exposure [39]. A study based on the data from NHANES III showed that the risk of metabolic syndrome, one of the cardiovascular risk factors, was increased by elevated cotinine levels in the participants aged 12−19 years, suggesting that there is a dose–response relationship with ETS exposure in adolescents as well [22].

The present study has some limitations. First, the definite cause and effect relationship could not be determined since it was a cross-sectional study. Second, the reference value for paediatric hs-CRP level has not been determined yet. Therefore, we adopted the reference range for adults from the AHA/CDC guidelines instead [21]. Third, serum cotinine, a biomarker of cigarette smoke exposure, was not available in the present study. Information on ETS exposure at home was assessed by a questionnaire completed by parents or guardians, and misclassification bias of parental reports depending on children’s health status could not be ruled out. However, there was no child having serious health problems. In a pilot study the KorEHS-C conducted in 2012 [19], the 6−11 year-old children’s cotinine levels in urine in those exposed to ETS were significantly higher than that in those who were not exposed to ETS. The geometric mean (geometric standard deviation) of cotinine level was 0.76 (3.17) and 0.35 (3.52) μg/L, respectively. This suggests little possibility of parental reporting bias in ETS exposure information. Fourth, there might have been residual confounding factors, such as family history of heart disease that we could not consider due to a lack of such information.

The present study was based on a nationally representative dataset of three to five year-old children and the results can therefore be generalized to Korean preschool children.

## 5. Conclusions

ETS exposure at home is associated with an increase in hs-CRP levels in three to five year-old children in a dose–response manner based on a Korean nationally representative dataset. Anti-smoking educational programs for parents and guardians may be helpful to reduce ETS exposure at home and to prevent the possible early onset of cardiovascular disease. The present findings need to be replicated by future studies with a larger sample size and longitudinal design.

## Figures and Tables

**Table 1:** General characteristics of the study subjects, Korean Environmental Health Survey in Children and Adolescents, 2014.

Variables	Total (*N* = 482)	ETS Non-Exposed (*n* = 412)	ETS Exposed (*n* = 70)	*p*-Value
Age (%)				
3 years	37.3 (33.9–40.9)	83.3 (72.8–90.3)	16.7 (9.7–27.2)	0.29
4 years	32.6 (29.6–35.7)	89.5 (83.7–93.4)	10.5 (6.6–16.3)	
5 years	30.1 (26.8–33.7)	83.0 (75.1–88.7)	17.0 (11.3–24.9)	
Sex (%)				
Male	51.8 (48.4–55.2)	86.7 (78.7–92.0)	13.3 (8.0–21.3)	0.47
Female	48.2 (44.8–51.6)	83.6 (77.4–88.4)	16.4 (11.6–22.6)	
Exposed to ETS at home (%)				
No	85.2 (80.3–89.1)			
Yes	14.8 (10.9–19.7)			
Frequency of ETS exposure at home per day (%)				
0	85.2 (80.3–89.1)			
1–2	11.5 (8.4–15.5)		77.9 (65.0–87.1)	
3–4	1.9 (0.8–4.2)		12.7 (6.4–23.6)	
≥5	1.4 (0.6–3.2)		9.4 (3.9–20.8)	
Body mass index (kg/m^2^)	16.21±0.10	16.23 ± 0.11	16.07 ± 0.32	0.60
White blood cell count (103/mL)	7.44 ± 0.16	7.45 ± 0.17	7.41 ± 0.30	0.89
Systolic blood pressure (mmHg)	96.00 ± 0.53	95.84 ± 0.59	96.96 ± 1.30	0.39
Diastolic blood pressure (mmHg)	61.03 ± 0.62	61.07 ± 0.66	60.79 ± 1.26	0.83
Fasting blood sugar (mg/dL)	91.32 ± 0.84	91.52 ± 0.83	90.15 ± 1.53	0.37
Total cholesterol (mg/dL)	159.54 ± 1.41	160.23 ± 1.63	155.60 ± 4.17	0.27
Hemoglobin (g/dL)	12.76 ± 0.05	12.76 ± 0.05	12.75 ± 0.11	0.92
History of asthma (%)				
No	94.0 (91.3–95.9)	85.7 (80.3–89.8)	14.3 (10.2–19.7)	0.40
Yes	6.0 (4.1–8.7)	78.3 (54.9–91.4)	21.7 (8.6–45.1)	
History of atopic dermatitis (%)				
No	70.6 (66.6–74.3)	87.9 (82.2–92.0)	12.1 (8.0–17.8)	0.04
Yes	29.4 (25.7–33.4)	79.7 (71.4–86.0)	20.3 (14.0–28.6)	
Household income level (%)				
Upper class	0.1 (0.0–1.1)	100	0.0	0.19
Upper-middle class	6.1 (3.9–9.5)	96.7 (79.6–99.5)	3.3 (0.5–20.4)	
Middle middle class	61.0 (55.2–66.5)	86.0 (79.4–90.7)	14.0 (9.3–20.6)	
Lower-middle class	29.1 (23.9–35.0)	82.4 (74.2–88.4)	17.6 (11.6–25.8)	
Lower class	3.6 (1.6–8.0)	72.3 (47.1–88.5)	27.7 (11.5–52.9)	

ETS: environmental tobacco smoke. Results shown as the mean±standard deviation for continuous variables and percentages (95% confidence intervals) for categorical variables. *p*-Value calculated using *t*-test or χ^2^-test for comparison between the ETS non-exposed and exposed. All analyses considered the complex sampling design. * Household income level based on the questionnaire.

**Table 2:** Level of serum high-sensitivity C-reactive protein according to general characteristics of study subjects, Korean Environmental Health Survey in Children and Adolescents, 2014.

Variables	High-Sensitivity C-Reactive Protein (mg/L)		*p*-Value
	Mean ± SD	Median (IQR)	
All	0.81 ± 0.07	0.38 (0.25–0.69)	
Age (years)			
3	0.82 ± 0.10	0.37 (0.24–0.69)	0.62
4	0.78 ± 0.10	0.38 (0.26–0.72)	
5	0.86 ± 0.11	0.38 (0.24–0.66)	
Sex			
Male	0.85 ± 0.10	0.37 (0.24–0.68)	0.47
Female	0.79 ± 0.08	0.39 (0.26–0.69)	
Exposed to ETS at home			
No	0.83 ± 0.08	0.37 (0.25–0.68)	0.81
Yes	0.73 ± 0.15	0.39 (0.23–0.78)	
Frequency of ETS exposure at home per day			
0	0.84 ± 0.07	0.37 (0.25–0.68)	0.59
1–2	0.58 ± 0.09	0.37 (0.24–0.63)	
3–4	1.08 ±0.31	0.94 (0.21–1.97)	
≥5	1.24 ± 0.58	0.72 (0.24–2.21)	
History of asthma			
No	0.83 ± 0.06	0.37 (0.25–0.69)	0.68
Yes	0.61 ± 0.11	0.44 (0.23–0.71)	
History of atopic dermatitis			
No	0.77 ± 0.07	0.37 (0.24–0.66)	0.26
Yes	0.93 ± 0.12	0.40 (0.25–0.80)	
Household income level *			
Upper class	0.402	0.40 (0.40–0.40)	0.75
Upper-middle class	0.59 ± 0.09	0.43 (0.24–0.90)	
Middle class	0.77 ± 0.07	0.37 (0.25–0.70)	
Lower-middle class	1.01 ± 0.15	0.37 (0.25–0.69)	
Lower class	0.48 ± 0.11	0.37 (0.21–0.46)	

ETS: environmental tobacco smoke; IQR: interquartile range; SD: standard deviation All analyses calculated considering the complex sampling design. *p*-Value calculated by the Kruskall-Wallis test. * Household income level based on the questionnaire.

**Table 3:** Association between ETS exposure at home and having high level of hs-CRP (≥1.0 mg/L).

ETS Exposure	No. of Children with hs-CRP ≥1.0 /Total	Crude PRR (95% CI)	Adjusted PRR1 (95% CI)	Adjusted PRR2 (95% CI)
Exposed to ETS at home				
No	69/412	reference	reference	reference
Yes	13/70	1.16 (0.53–2.52)	1.22 (0.56–2.63)	1.28 (0.59–2.76)
Frequency of ETS exposure at home a day				
0	69/412	reference	reference	reference
1–2	6/56	0.48 (0.15–1.52)	0.50 (0.16–1.55)	0.52 (0.17–1.66)
3–4	4/8	4.11 (0.90–18.75)	4.93 (1.05–23.08)	4.90 (1.04–23.17)
≥5	3/6	10.09 (1.68–60.67)	10.15 (2.05–50.35)	11.66 (1.90–71.65)
*p* for trend		0.06	0.04	0.03

hs-CRP: high sensitivity C-reactive protein; ETS: Environmental tobacco smoke. Prevalence rate ratio (PRRs) and 95% Confidence Intervals (CIs) estimated using survey logistic regression. Adjusted PRR 1 included age, gender, body mass index; adjusted PRR2 included age, gender, body mass index, white blood cell count, history of asthma, history of atopic dermatitis as covariates in the multivariate models. *p* for trend calculated using the continuous scale of the categorical variable in the corresponding model.

## Supplementary Materials

The following are available online at www.mdpi.com/1660-4601/14/10/1105/s1, Table S1: General characteristics of the study subjects and the excluded.
