# Supplementary material for: Environmental Tobacco Smoke Exposure at Home and High-Sensitivity C-Reactive Protein Levels in Three-to-Five-Year-Old Children
Source: Int J Environ Res Public Health. 2017 Sep 23;14(10):1105. doi: 10.3390/ijerph14101105 (PMC5664606; doi:10.3390/ijerph14101105)
Supplement: Supplementary file 1 [file ijerph-14-01105-s001.pdf]

Supplementary Table 1. General characteristics of the study subjects and the excluded.

Variables	Included (n=482)	Excluded (n=95)	p Value
Age (years)	3.93±0.12	4.24±0.10	<0.05
Sex			
Male	51.8 (48.4-55.2)	51.4 (41.0-61.6)	0.94
Female	48.2 (44.8-51.6)	48.6 (38.4-59.0)	
Exposed to SHS at home			
No	85.2 (80.3-89.1)	77.7 (58.9-89.5)	0.33
Yes	14.8 (10.9-19.7)	22.3 (10.5-41.1)	
Frequency of SHS exposure at home a day			
0	85.2 (80.3-89.1)	77.7 (58.9-89.5)	0.27
1-2	11.5 (8.4-15.5)	21.4 (9.8-40.5)	
3-4	1.9 (0.8-4.2)	0.9 (0.1-6.2)	
≥5	1.4 (0.6-3.2)	0.0	
Body mass index (kg/m2)	16.21±0.10	16.02±0.14	0.23
White blood cell count (10^3/ml)	7.44±0.16	7.64±0.17	0.32
Systolic blood pressure (mmHg)	96.00±0.53	96.99±1.30	0.50
Diastolic blood pressure (mmHg)	61.03±0.62	61.65±1.45	0.67
Fasting blood sugar (mg/dl)	91.32±0.84	91.74±1.65	0.81
Total cholesterol (mg/dl)	159.54±1.41	161.97±4.00	0.59
Hemoglobin (g/dL)	12.76±0.05	12.79±0.10	0.81
History of asthma			
No	94.0 (91.3-95.9)	91.9 (81.3-96.7)	0.53
Yes	6.0 (4.1-8.7)	8.1 (3.3-18.7)	
History of atopic dermatitis			
No	70.6 (66.6-74.3)	64.8 (52.9-75.1)	0.29
Yes	29.4 (25.7-33.4)	35.2 (24.9-47.1)	
Economic status of family			
Upper class	0.1 (0.0-1.1)	0.0	0.80
Upper of middle class	6.1 (3.9-9.5)	3.6 (0.9-13.2)	
Middle of middle class	61.0 (55.2-66.5)	56.1 (41.3-69.9)	
Lower of middle class	29.1 (23.9-35.0)	36.1 (23.6-50.9)	
Lower class	3.6 (1.6-8.0)	4.2 (1.0-15.3)	
